# Supplementary material for: Real world data on cervical cancer treatment patterns, healthcare access and resource utilization in the Brazilian public healthcare system
Source: PLoS One. 2024 Oct 30;19(10):e0312757. doi: 10.1371/journal.pone.0312757 (PMC11524504; doi:10.1371/journal.pone.0312757)
Supplement: S1 Table — *From first claim of C53 to treat procedure (surgery/CT/RT), except conization. (DOCX) [file pone.0312757.s001.docx]

**Supplementary Table 1.** Time to treatment initiation according to disease stage and country region, from staged cohort.

|  | **Brazil** | **North** | **Northeast** | **Midwest** | **Southeast** | **South** |
| --- | --- | --- | --- | --- | --- | --- |
| **N** | **90,073** | **9,204 (10.2)** | **27,371 (30.4)** | **6,667 (7.4)** | **31,821 (35.3)** | **15,010 (16.7)** |
| **Time to initiate treatment*** (months) | |  |  |  |  |  |
| ***Non-advance stage*** |  |  |  |  |  |  |
| ***Stage I+II*** | **32,345** | **4,344** | **9,579** | **2,405** | **11,134** | **4,883** |
| Mean (SD) | 4.16 (8.93) | 4.19 (8.92) | 3.81 (8.18) | 3.97 (8.79) | 4.48 (9.3) | 4.17 (9.48) |
| Median (IQR) | 1.02 (1.02 - 3.02) | 1.02 (1.02 - 3.02) | 1.02 (1.02 - 2.98) | 1.02 (0.98 - 2.92) | 1.02 (1.02 - 3.02) | 1.02 (0.98 - 2.95) |
| Strata - N (%) |  |  |  |  |  |  |
| <1 m | 7,847 (24.3) | 1,064 (24.5) | 2,332 (24.3) | 647 (26.9) | 2,523 (22.7) | 1,281 (26.2) |
| 1-<2 m | 14,692 (45.4) | \| 1,912 (44.0) \| \| --- \| | 4,381 (45.7) | 1,116 (46.4) | 5,003 (44.9) | 2,280 (46.7) |
| 2-<3 m | 1,425 (4.4) | 168 (3.9) | 494 (5.2) | 90 (3.7) | 488 (4.4) | 185 (3.8) |
| 3-<6 m | 4,213 (13.0) | 603 (13.9) | 1,279 (13.4) | 271 (11.3) | 1,495 (13.4) | 565 (11.6) |
| 6-<12 m | 1,902 (5.9) | 302 (7.0) | 520 (5.4) | 111 (4.6) | 747 (6.7) | 222 (4.5) |
| 12+ m | 2,266 (7.0) | 295 (6.8) | 573 (6.0) | 170 (7.1) | 878 (7.9) | 350 (7.2) |
| *Missing* | *3,009* | *224* | *764* | *213* | *1,356* | *452* |
|  |  |  |  |  |  |  |
| **Stage I** | **9,236** | **718** | **2,472** | **720** | **3,490** | **1,836** |
| Mean (SD) | 4.63 (8.99) | 5.22 (9.69) | 4.21 (8.07) | 4.27 (8.39) | 5.08 (9.45) | 4.25 (9.19) |
| Median (IQR) | 1.02 (1.02 - 4.0) | 1.02 (1.02 - 5.02) | 1.02 (1.02 - 3.97) | 1.02 (1.02 - 3.02) | 1.02 (1.02 - 4.03) | 1.02 (1.02 - 3.02) |
| Strata - N (%) |  |  |  |  |  |  |
| <1 m | 1,987 (21.5) | 153 (21.3) | 523 (21.2) | 159 (22.1) | 700 (20.1) | 452 (24.6) |
| 1-<2 m | 3,885 (42.1) | 270 (37.6) | 1,040 (42.1) | 333 (46.2) | 1,416 (40.6) | 826 (45.0) |
| 2-<3 m | 432 (4.7) | 28 (3.9) | 137 (5.5) | 22 (3.1) | 164 (4.7) | 81 (4.4) |
| 3-<6 m | 1,441 (15.6) | 125 (17.4) | 410 (16.6) | 105 (14.6) | 561 (16.1) | 240 (13.1) |
| 6-<12 m | 742 (8.0) | 74 (10.3) | 197 (8.0) | 47 (6.5) | 317 (9.1) | 107 (5.8) |
| 12+ m | 749 (8.1) | 68 (9.5) | 165 (6.7) | 54 (7.5) | 332 (9.5) | 130 (7.1) |
| *Missing* | *1,381* | *40* | *298* | *95* | *708* | *240* |
|  |  |  |  |  |  |  |
| **Stage II** | **23,109** | **3,626** | **7,107** | **1,685** | **7,644** | **3,047** |
| Mean (SD) | 3.97 (8.89) | 3.99 (8.75) | 3.67 (8.21) | 3.84 (8.95) | 4.21 (9.23) | 4.13 (9.65) |
| Median (IQR) | 1.02 (0.98 - 2.98) | 1.02 (0.98 - 3.02) | 1.02 (0.98 - 2.95) | 1.02 (0.98 - 2.0) | 1.02 (1.02 - 2.98) | 1.02 (0.98 - 2.03) |
| Strata - N (%) |  |  |  |  |  |  |
| <1 m | 5,860 (25.4) | 911 (25.1) | 1,809 (25.5) | 488 (29.0) | 1,823 (23.8) | 829 (27.2) |
| 1-<2 m | 10,807 (46.8) | 1,642 (45.3) | 3,341 (47.0) | 783 (46.5) | 3,587 (46.9) | 1,454 (47.7) |
| 2-<3 m | 993 (4.3) | 140 (3.9) | 357 (5.0) | 68 (4.0) | 324 (4.2) | 104 (3.4) |
| 3-<6 m | 2,772 (12.0) | 478 (13.2) | 869 (12.2) | 166 (9.9) | 934 (12.2) | 325 (10.7) |
| 6-<12 m | 1,160 (5.0) | 228 (6.3) | 323 (4.5) | 64 (3.8) | 430 (5.6) | 115 (3.8) |
| 12+ m | 1,517 (6.6) | 227 (6.3) | 408 (5.7) | 116 (6.9) | 546 (7.1) | 220 (7.2) |
| *Missing* | *1,628* | *184* | *466* | *118* | *648* | *212* |
|  |  |  |  |  |  |  |
| ***Advanced stage*** |  |  |  |  |  |  |
| ***Stage III+IV*** | **51,187** | **4,383** | **15,967** | **3,756** | **17,982** | **9,099** |
| Mean (SD) | 4.12 (9.27) | 3.99 (8.64) | 3.88 (8.78) | 4.26 (9.68) | 4.36 (9.61) | 4.08 (9.5) |
| Median (IQR) | 1.02 (0.98 - 2.95) | 1.02 (0.98 - 2.95) | 1.02 (1.02 - 2.95) | 1.02 (0.98 - 2.03) | 1.02 (1.02 - 2.98) | 1.02 (0.98 - 2.0) |
| Strata - N (%) |  |  |  |  |  |  |
| <1 m | 12,941 (25.3) | 1,123 (25.6) | 3,889 (24.4) | 997 (26.5) | 4,484 (24.9) | 2,448 (26.9) |
| 1-<2 m | 24,478 (47.8) | 2,048 (46.7) | 7,726 (48.4) | 1,809 (48.2) | 8,388 (46.6) | 4,507 (49.5) |
| 2-<3 m | 2,100 (4.1) | 174 (4.0) | 711 (4.5) | 125 (3.3) | 749 (4.2) | 341 (3.7) |
| 3-<6 m | 5,577 (10.9) | 491 (11.2) | 1,904 (11.9) | 351 (9.3) | 2,061 (11.5) | 770 (8.5) |
| 6-<12 m | 2,366 (4.6) | 242 (5.5) | 707 (4.4) | 184 (4.9) | 890 (4.9) | 343 (3.8) |
| 12+ m | 3,725 (7.3) | 305 (7.0) | 1,030 (6.5) | 290 (7.7) | 1,410 (7.8) | 690 (7.6) |
| *Missing* | *3,532* | *253* | *1,061* | *293* | *1,349* | *576* |
|  |  |  |  |  |  |  |
| **Stage III** | **33,407** | **2,946** | **11,654** | **2,417** | **11,248** | **5,142** |
| Mean (SD) | 3.84 (8.76) | 3.61 (7.86) | 3.6 (8.12) | 3.72 (8.69) | 4.14 (9.35) | 3.92 (9.31) |
| Median (IQR) | 1.02 (0.98 - 2.03) | 1.02 (0.98 - 2.92) | 1.02 (1.02 - 2.92) | 1.02 (0.98 - 2.0) | 1.02 (1.02 - 2.98) | 1.02 (0.98 - 2.0) |
| Strata - N (%) |  |  |  |  |  |  |
| <1 m | 8,466 (25.3) | 781 (26.5) | 2,797 (24.0) | 681 (28.2) | 2,796 (24.9) | 1,411 (27.4) |
| 1-<2 m | 16,195 (48.5) | 1,377 (46.7) | 5,734 (49.2) | 1,153 (47.7) | 5,335 (47.4) | 2,596 (50.5) |
| 2-<3 m | 1,409 (4.2) | 120 (4.1) | 531 (4.6) | 82 (3.4) | 476 (4.2) | 200 (3.9) |
| 3-<6 m | 3,765 (11.3) | 340 (11.5) | 1,447 (12.4) | 242 (10.0) | 1,340 (11.9) | 396 (7.7) |
| 6-<12 m | 1,428 (4.3) | 157 (5.3) | 493 (4.2) | 113 (4.7) | 499 (4.4) | 166 (3.2) |
| 12+ m | 2,144 (6.4) | 171 (5.8) | 652 (5.6) | 146 (6.0) | 802 (7.1) | 373 (7.3) |
| *Missing* | *2,180* | *163* | *735* | *161* | *813* | *308* |
|  |  |  |  |  |  |  |
| **Stage IV** | **17,780** | **1,437** | **4,313** | **1,339** | **6,734** | **3,957** |
| Mean (SD) | 4.64 (10.13) | 4.76 (10.01) | 4.64 (10.33) | 5.22 (11.2) | 4.71 (10.01) | 4.3 (9.74) |
| Median (IQR) | 1.02 (0.98 - 2.98) | 1.02 (1.02 - 3.02) | 1.02 (0.98 - 2.98) | 1.02 (1.02 - 2.98) | 1.02 (0.98 - 3.02) | 1.02 (0.98 - 2.03) |
| Strata - N (%) |  |  |  |  |  |  |
| <1 m | 4,475 (25.2) | 342 (23.8) | 1,092 (25.3) | 316 (23.6) | 1,688 (25.1) | 1,037 (26.2) |
| 1-<2 m | 8,283 (46.6) | 671 (46.7) | 1,992 (46.2) | 656 (49.0) | 3,053 (45.3) | 1,911 (48.3) |
| 2-<3 m | 691 (3.9) | 54 (3.8) | 180 (4.2) | 43 (3.2) | 273 (4.1) | 141 (3.6) |
| 3-<6 m | 1,812 (10.2) | 151 (10.5) | 457 (10.6) | 109 (8.1) | 721 (10.7) | 374 (9.5) |
| 6-<12 m | 938 (5.3) | 85 (5.9) | 214 (5.0) | 71 (5.3) | 391 (5.8) | 177 (4.5) |
| 12+ m | 1,581 (8.9) | 134 (9.3) | 378 (8.8) | 144 (10.8) | 608 (9.0) | 317 (8.0) |
| *Missing* | *1,352* | *90* | *326* | *132* | *536* | *268* |
| *From first claim of C53 to treat procedure (surgery/CT/RT), except conization | | | | | | |
