# Supplementary material for: Real world data on cervical cancer treatment patterns, healthcare access and resource utilization in the Brazilian public healthcare system
Source: PLoS One. 2024 Oct 30;19(10):e0312757. doi: 10.1371/journal.pone.0312757 (PMC11524504; doi:10.1371/journal.pone.0312757)
Supplement: S2 Table — *(Euclidean) distance is calculated based on the city of residence and of the institution (latitude and longitude). 1 Number of visits to the health center categorized by distance. The percentage reflects the proportion of the total number of visits. (DOCX) [file pone.0312757.s002.docx]

**Supplementary Table 2.** Distance from residence to healthcare facility, per visit.

|  | **All patient** | | | **Non-advanced** | | | **Advanced** | | |
| --- | --- | --- | --- | --- | --- | --- | --- | --- | --- |
|  |  |  |  | **Stage I** | | **Stage II** | **Stage III** | | **Stage IV** |
|  | 90,073 (100) | | | 10,617 (5.1) | | 24,737 (12) | 35,587 (17.2) | | 19,132 (9.2) |
| **Distance from residence to healthcare facility, per visit*** (km) | |  |  | |  | | |  |  |
| Mean (SD) | 112.09 (549.62) | | | 108.49 (550.48) | | 144.91 (658.67) | 127.03 (565.79) | | 135.64 (623.74) |
| Median (IQR) | 0.0 (0.0 - 61.9) | | | 0.0 (0.0 - 60.37) | | 0.0 (0.0 - 95.15) | 7.96 (0.0 - 89.16) | | 4.88 (0.0 - 75.16) |
| Strata (km)^1^, N (%) |  | | |  | |  |  | |  |
| ≤50 | 3,252,089 (72.6) | | | 236,915 (72.6) | | 509,806 (67.2) | 752,394 (67.5) | | 465,935 (69.9) |
| 51-100 | 355,001 (7.9) | | | 30,421 (9.3) | | 64,498 (8.5) | 98,644 (8.9) | | 58,214 (8.7) |
| 101-200 | 436,655 (9.7) | | | 29,338 (9.0) | | 90,486 (11.9) | 137,931 (12.4) | | 72,669 (10.9) |
| 201-500 | 249,937 (5.6) | | | 17,255 (5.3) | | 54,147 (7.1) | 74,622 (6.7) | | 37,875 (5.7) |
| 501-750 | 55,694 (1.2) | | | 3,554 (1.1) | | 10,741 (1.4) | 14,102 (1.3) | | 7,202 (1.1) |
| 750-1,000 | 24,073 (0.5) | | | 1,623 (0.5) | | 4,216 (0.6) | 5,835 (0.5) | | 4,407 (0.7) |
| 1,001-1,500 | 31,513 (0.7) | | | 2,242 (0.7) | | 6,732 (0.9) | 8,826 (0.8) | | 5,068 (0.8) |
| > 1,500 | 74,820 (1.7) | | | 5,015 (1.5) | | 18,180 (2.4) | 21,531 (1.9) | | 15,447 (2.3) |
| *(Euclidean) distance is calculated based on the city of residence and of the institution (latitude and longitude)  ^1^ Number of visits to the health center categorized by distance. The percentage reflects the proportion of the total number of visits. | | | | | | | | | |
