# Supplementary material for: Real world data on cervical cancer treatment patterns, healthcare access and resource utilization in the Brazilian public healthcare system
Source: PLoS One. 2024 Oct 30;19(10):e0312757. doi: 10.1371/journal.pone.0312757 (PMC11524504; doi:10.1371/journal.pone.0312757)
Supplement: S3 Table — *Patients may undergo multiple surgical procedures. **Surgery: hysterectomy or trachelectomy (if no hysterectomy procedure). *** A total of 90,073 patients had stage information available. (DOCX) [file pone.0312757.s003.docx]

**Supplementary Table 3.** Cervical cancer treatment according to disease stage.

|  | *****Non-advanced** | | *****Advanced** | |
| --- | --- | --- | --- | --- |
|  | **Stage I** | **Stage II** | **Stage III** | **Stage IV** |
| **N** | 10,617 (7.1) | 24,737 (16.6) | 35,587 (23.9) | 19,132 (12.8) |
| **Therapy - N (%)** |  |  |  |  |
| Surgery only | - | - | - | - |
| Surgery + RT | 1,880 (17.7) | 1,493 (6.0) | 1,334 (3.7) | 562 (2.9) |
| Surgery + CT | 285 (2.7) | 590 (2.4) | 988 (2.8) | 1,136 (5.9) |
| Surgery + CRT | 1,418 (13.4) | 5,270 (21.3) | 7,774 (21.8) | 2,737 (14.3) |
| RT only | 3,741 (35.2) | 4,206 (17.0) | 4,888 (13.7) | 2,299 (12.0) |
| CT only | 799 (7.5) | 2,069 (8.4) | 3,882 (10.9) | 5,253 (27.5) |
| CRT | 2,494 (23.5) | 11,109 (44.9) | 16,721 (47.0) | 7,145 (37.3) |
| ***Per type*** |  |  |  |  |
| **Surgery^*^ - N (%)** |  |  |  |  |
| **Cervical treat** | 3,579 (33.7) | 7,350 (29.7) | 10,090 (28.4) | 4,434 (23.2) |
| Cone | 2,071 (57.9) | 5,303 (72.1) | 7,382 (73.2) | 2,969 (67.0) |
| Only one | 1,690 (81.6) | 4,327 (81.6) | 6,083 (82.4) | 2,513 (84.6) |
| 1+ | 381 (18.4) | 976 (18.4) | 1,299 (17.6) | 456 (15.4) |
| Trachelectomy | 69 (1.9) | 161 (2.2) | 232 (2.3) | 120 (2.7) |
| Hysterectomy | 656 (18.3) | 1,165 (15.9) | 1,491 (14.8) | 793 (17.9) |
| Hysterectomy with pelvic exenteration | 1,505 (42.1) | 1,795 (24.4) | 2,417 (24.0) | 1,128 (25.4) |
| **Lymphadenectomy** | 24 (0.23) | 45 (0.18) | 84 (0.24) | 35 (0.18) |
| Pelvic | 21 (87.5) | 18 (40.0) | 23 (27.38) | 11 (31.43) |
| Retroperitoneal | 2 (8.33) | 21 (46.67) | 41 (48.81) | 13 (37.14) |
| Other | 1 (4.17) | 6 (13.33) | 20 (23.81) | 12 (34.29) |
| **Radiotherapy - N (%)** | *2,028 (19.1)* | *5,315 (21.5)* | *7,329 (20.6)* | *2,928 (15.3)* |
| External | 1 508 (74.4) | 4 332 (81.5) | 6 232 (85.0) | 2 664 (91.0) |
| Brachytherapy | 1 431 (70.6) | 3 890 (73.2) | 4 709 (64.3) | 1 368 (46.7) |
| ***RT or CT before and/or after surgical procedure*** | |  |  |  |
| **Radiotherapy - N (%)** | 1,998 (18.82) | 2,635 (10.65) | 3,400 (9.55) | 1,367 (7.15) |
| Before surgery** | 327 (16.4) | 984 (37.3) | 1,216 (35.8) | 387 (28.3) |
| After surgery** | 1,717 (85.9) | 1,751 (66.5) | 2,268 (66.7) | 1,011 (74.0) |
| **Chemotherapy - N (%)** | 828 (7.8) | 2,164 (8.75) | 3,288 (9.24) | 1,684 (8.8) |
| Before surgery** | 257 (31.0) | 991 (45.8) | 1,323 (40.2) | 539 (32.0) |
| After surgery** | 651 (78.6) | 1,381 (63.8) | 2,293 (69.7) | 1,271 (75.5) |

*Patients may undergo multiple surgical procedures

**Surgery: hysterectomy or trachelectomy (if no hysterectomy procedure)

*** A total of 90,073 patients had stage information available.
