# Supplementary material for: Real world data on cervical cancer treatment patterns, healthcare access and resource utilization in the Brazilian public healthcare system
Source: PLoS One. 2024 Oct 30;19(10):e0312757. doi: 10.1371/journal.pone.0312757 (PMC11524504; doi:10.1371/journal.pone.0312757)
Supplement: Attachment — Submitted filename: Review Plos One 2024.docx [file pone.0312757.s004.docx]

Hello, Thabata! I hope this finds you well. Good luck!

1. Line 26: “Data from January-2014 until December-2020”. Consider replace ‘until’ with ‘to’.
2. Lines 38-39: “The treatment pattern showed that chemoradiotherapy was the **more** frequent regimen 39 overall and that the use of chemotherapy increas**es** with staging, as well as the HCRU.”
   - Suggestion: The treatment pattern showed that chemoradiotherapy was the **most** frequent regimen overall and the use of chemotherapy and HCRU increas**ed** with staging.
3. Lines 56-57: “has been available for girls from 9 to 14 years since 2014 and 57 for boys from 11 to 14 years since 2017” consider replacing with “has been available for girls aged 9 to 14 e for boys aged 11 to 14” or “has been available for girls from 9 to 14 years old since 2014 and for boys from 11 to 14 years old since 2017 “
4. Line 59: “the most effective exam to detect precancerous abnormalities and prevent CC” – reference?
   - There are recent studies that recognize the HPV DNA or pap smear + HPV DNA as the most effective exam to detect precancerous abnormalities.
5. Line 61: “in case of normal results” – consider replacing with “negative results”. What is a normal result?
6. Line 63: “Despite national programs for CC cancer prevention” – the word ‘cancer’ is written twice. ‘CC’ (cervical cancer) and ‘carcer’ right after.
7. Line 68: I missed mentioning brachytherapy as a line of treatment.
8. Line 78: “between regions of the country related to social, economic and health access determinants” – perhaps you should also include ‘geographical’
9. Line 79: “decreased efficacy in the detection of disease” be aware of the semantic differences between effectiveness and efficacy
10. Line 80: “To create strategies to reduce the CC burden in Brazil” - It’s more common for native speakers to start a sentence with the gerund as subject than the infinitive form, like “creating strategies to reduce… “
11. Line 90: “SUS is the universal healthcare system of Brazil, which covers 75% of the population [23]”. Is this a necessary information? Is this a precise information? If the SUS is the ‘universal’ healthcare, how can it cover only 75%? SUS is widely available to all the population. It’s a constitutional right. The fact that 24% of users possess private healthcare coverage doesn’t mean they don’t use the system in some way.
12. Line 105: “Data **from** January 2014 **until** December 2020 was gathered of all patients with the 106 International Classification of Diseases tenth revision for cervical cancer (ICD-10 code C53)”. The reference?
    - Consider replace ‘until’ with ‘to’.
13. Line 124: Consider replace ‘until’ with ‘to’. You have already said the study’s period. Maybe you could replace the ‘data from … to… ‘ with another expression.
14. Line 130: “Patient’s stages were considered in the first ICD-10 claim in SIA-AQ and SIA-AR databases (subsets of chemotherapy and radiotherapy from SIA database), which provides stage information.” In the same sentence, you have said that those databases provide stage information twice. Also, you have said that previously in lines 102-103.
15. Lines 130-132: “Patients with stage inconsistent information (i.e., stage information different from I to IV) at first cervical cancer claim were excluded (Fig 1)” – It would be better written as ‘patients with inconsistent stage information…’
16. Line 139: “Additionally, in the staged patient’s distance”. Take the ‘in’ word out.
17. Lines 140-142: “(measured as the date of treatment initiation, defined in this study as the time elapsed from the first claim of ICD142 10 C53 to first treatment procedure [CT, RT or surgery – except conization/cone biopsy])”.
    - Consider removing the ‘measured as the date of treatment initiation’ and just use the ‘defined in this study…’. They explain the same thing, it’s repetitive.
18. Lines 149-150: “with absolute frequencies and percentages with 95% confidence interval (CI) and percentages”. You’ve written ‘percentages’ twice.
19. Lines 153-154: “Thus, the longer the follow-up, lower the PPPM will be, even if there are many procedures claims”. You don’t need the ‘will be’, because you are already using a comparative form.
20. Line 157: “The number of LOTs received was summarized.” – I understand this information was already stated in the previous sentence “…and most frequently reported treatments for each line of treatment (LOT) using a Sankey diagram”.
21. Are the abbreviations ‘pppm’ and ‘hcru’ official or used in other reference? They’re confusing and difficult to associate along the reading. I had to return to the Methods section all the time to understand them.
22. Table 1
    - In the title: ‘Description of sociodemographic characteristics of included patients’ instead of ‘(…)patients included’.
    - In ‘Ethnicity N(%)’ the percentage and the absolute numbers doesn’t match with the total included population (206,861). After several calculations, I concluded that the percentages were calculated after the ‘all patients’ minus ‘missing information’ and that’s not clear in the text. Meaning, in this case, the total N is not 206,861, but 178,582. Is that correct? Perhaps you could add an asterisk to clear that data like you did in ‘outpatient visit’ in table 2.
    - You have already said in the Methods section about the age at index date, and in the Results section about the total of staged patients. Is it necessary to write that again in the table?
23. Table 2:
    - Hospitalization rate in days???
    - “Outpatient visit^1^” – What does that superscripted number refer to?
    - Line 201: “* Includes all patients with stage 1-4, and those non-staged.”. I didn’t find any asterisk in the table. I guess this note refers to the “Outpatient visit^1^”.
    - Is there any information/data on brachytherapy?
    - You have already said in the Results section about the total of staged patients. Is it necessary to write that again in the table?
24. Line 199-200: “Legend: LOS: length of stay; IQR: interquartile range; CI: confidence interval; SD: standard 200 deviation; PPPM: per patient per month.” Those abbreviations were already explained previously.
25. Line 250 and S2 table: “Patients with stage III and IV have traveled a greater median distance of 7.96 km [IQR 0.0 - 89.16] compared to 4.88 km 252 [IQR 0.0 - 75.16] in the non-advanced stages (S2 Table)” However, in the Supplementary table, ‘4.88 km 252 [IQR 0.0 - 75.16]’ refers to advanced stage IV. You should review the table later.
26. S2 Table: The stratification per kilometers is the sum of all the kilometers spent traveling by all the patients on all the visits, correct? I took some time to get that.
27. S3 Table:
    - ‘Surgery^3^ - N (%)’ – What does the superscripted number stand for?
    - You don’t need the comma in the sentence ‘Cervical cancer treatment, according to disease stage.’
28. Line 259: ‘Lymphadenectomy with pelvic lymphadenectomy’. Try to replace for only ‘pelvic lymphadenectomy’’
29. Line 268: “About 90% of stage I patients versus 67% of stage IV patients were submitted to RT (Error! Reference source not found.).” I don’t think there’s any reference in there.
30. Line 295: Avoid using the first-person plural. Suggestion: ‘so far’.
31. Line 297: ‘Although there were improvements in the prevention of cervical cancer in Brazil’. The prevention policies continue to improve, perhaps you should write as: “Although there have been improvements… “
32. Line 299: “Like our results” –Avoid using the first-person plural. Suggestion: ‘Similar to these findings, other Brazilian studies’
33. Line 300: mean or median?
34. Line 301: ‘were’ not ‘are’
35. Line 324: Replace ‘in most wealthy’ with ‘in wealthier’
36. Line 324: “what was mentioned about health access and population’s well-being” – mentioned where? What about health access and population’s well-being?
37. Line 325-326: “as with higher income per-capita” – ‘per-capita income’
38. Lines 325-327: “since these regions are characterized as with higher income per-capita, educational levels, concentration of health services, access to reference medical units and higher health resources per-capita in both private and public sectors in these regions” – ‘these regions’ is mentioned twice in the same sentence. Perhaps you should consider turn this sentence into two different ones, so it doesn’t get confused.
39. ‘higher health resources per-capita’ 🡪 ‘higher per-capita health resources’
40. Lines 336-339: Suggestion: “Even though it has been mandatory for all cancer patients to start treatment within 60 days from the confirmed histological diagnosis, this analysis shows that it is not yet a reality for all patients diagnosed with CC”.
41. Lines 351-354: This is confusing – “In patients with recurrent, persistent, or metastatic disease paclitaxel regimens, in addition to cisplatin and other drugs, and associated to RT, are usually used in the clinical practice in patients with advancing disease treated under SUS, which was observed in this study”
    - Suggestion: In patients with recurrent, persistent, or metastatic disease paclitaxel regimens associated to cisplatin or other drugs and RT are commonly used in the clinical practice, as observed in this study.
42. Lines 355-358: The verb tenses are confused in this sentence, because It’s a finished action in a specific time of the past, so we use the simple past not the present perfect. “In 2021, however, new evidence has shown that adjuvant chemotherapy given after standard cisplatin-based chemoradiation for women with locally advanced CC has no effect in overall survival or progression free survival, changing the management of the disease”
    - Suggestion: In 2021, however, new evidence showed that adjuvant chemotherapy given after standard cisplatin-based chemoradiation for women with locally advanced CC had no effect in overall survival or progression free survival, changing the management of the disease.
43. Line 371: Avoid using the first-person plural – Suggestion: ‘Although this study…’
44. Lines 384-390: “. Furthermore, the HPV vaccine has been available in SUS since 2014, initially for girls aged 385 11 to 13 years, with a gradual expansion to other age groups, being currently recommended for 386 girls in the age group from 9 to 14 years, and for boys aged 11 to 14 years. However, immediate effects of HPV vaccination could not be seen since the target population is consisted of younger age groups and the disease usually appears with the advancing age, indicating the need of surveillance of the long-term effects and herd immunity, and an improvement of treatment standards and outcomes in the country”
    - You have mentioned this vaccine information previously in the Background section. It’s not necessary repeat it.
    - “Could” is commonly used to describe potential or hypothetical situations. I don’t believe it fits in this sentence very well
    - Suggestion: Despite HPV vaccine has been available in SUS since 2014, its immediate effects cannot yet be observed, as the target population consists of younger age groups, and the disease usually appears later in life, indicating the need for effects and herd immunity long-term surveillance, as well as improvements in treatment standards and outcomes in the country.
45. Line 399-400 – Suggestion: “Patient may undergo to Conization procedure in an initial stage; however, he’s only included in SIA-AQ and SIA-AR databases after an advanced stage diagnosis.”
46. Lines 408-409: It’s not correct to assume that private healthcare users do not use the public health system. We don’t know for sure the percentage of the Brazilian population that certainly use the public health system. So, you’d better change this last sentence.
47. In the limitations setting, probably you should write about the fact that those are secondary data and the underreporting of data related to the database in a country of continental dimensions and with significant deficiencies in the public health system. In addition, you should highlight about the need for more complete and efficient database, as well as the need for better methods to collect and work this type of data, given its importance for public health service.
