# Supplementary material for: Real world data on cervical cancer treatment patterns, healthcare access and resource utilization in the Brazilian public healthcare system
Source: PLoS One. 2024 Oct 30;19(10):e0312757. doi: 10.1371/journal.pone.0312757 (PMC11524504; doi:10.1371/journal.pone.0312757)
Supplement: Attachment — Submitted filename: The PLOS manuscript review.docx [file pone.0312757.s005.docx]

**The manuscript by Campuzano and collaborators, presenting an analysis on “Real world data on cervical cancer treatment patterns, healthcare access and resource utilization in the Brazilian public health system”, deserves publication.**

In general, the manuscript provides an interesting and useful analysis of a large population, derived from the Brazilian national data, based on healthcare records accumulated over 7 years (2014-2020), and offers evidence to refining the public health policy regarding access to cervical cancer treatment, lines of treatment, and resources utilization.

Still, the paper can be further improved for clarity by amendments in the text (and grammar). Also the references need further attention to quoting more recent, more relevant documents, and for accuracy, in quoting completed references, as some references lack journal names or records, such as page numbers, or sometimes quote the wrong page number. Please add the links to the webpages (url) where the references can be easily accessed, as possible.

Specific points for improvements:

Line 49: the number of 6,627 deaths cannot be found in the “Brasil. Ministério da saúde. Instituto Nacional de Câncer. Estimativa de Câncer no Brasil. 2020”, but rather on a news article of 2023 available at <https://agenciabrasil.ebc.com.br/saude/noticia/2023-07/diagnosticos-de-cancer-no-colo-do-utero-devem-somar-17-mil-ate-2025> .

However, the scientific reference mentioning the number of 6,627 deaths can be found on the article “Cervical cancer screening, treatment, and prophylaxis in Brazil: Current and future perspectives for cervical cancer elimination. Flávia M. Corrêa, Arn Migowski, Liz M. de Almeida, and Marcelo A. Soares. Frontiers of Medicine (Lausanne). 2022; 9: 945621.Published online 2022 Aug 24. doi: 10.3389/fmed.2022.945621 PMCID: PMC9449345 PMID: 36091685

I invite the authors to ascertain and validate the source of data, and replace reference 3 by F Correa et al. 2022.

Line 50: replace the word “expected” by “reported” for 2020, as the number of deaths was so reported in the above reference.

Line 50: I could not find the number of “20.48” on the paper by Santos MDO, Fernando J, Oliveira P, Maria L, Almeida D, Cancela MDC. Estimativa de Incidência de Câncer no Brasil, 2023-2025.

It seems that 20.48 cases per 100.000 women relates to incidence - not prevalence - of type of cancer: the publication mentioning this figure is by Flavia Correa et al., 2022. This number can also be found on the online article of INCA of 2023, please see reference below: harmonize references accordingly. <https://www.gov.br/inca/pt-br/assuntos/gestor-e-profissional-de-saude/controle-do-cancer-do-colo-do-utero/dados-e-numeros/incidencia>

Line 52: as mentioned above, I suggest indicate reference 3 (as above), not 2.

Line 53: please update the WHO statement and mention that WHO adopted in 2020 the “Global strategy to accelerate the elimination of cervical cancer as a public health problem”, based on three pillars : vaccination, screening, and treatment, as priority to reduce morbidity and mortality, as well as costs related to cervical cancer.

Line 55: the better and more recent reference no. 4 should be the WHO Global strategy to accelerate the elimination of cervical cancer as a public health problem. 2020, cf. <https://www.who.int/publications/i/item/9789240014107>

Line 57: please add one sentence to clarify that today HPV vaccination coverage in Brazil remains low, as only less than 10% of young women 19-24 years old have been vaccinated.

Line 58: rephrase for clarity that In Brazil until today the CC screening program has been based on cytology by Papanicolau smear (Pap smear), for women aged 25 to 64 years old.

Line 60: please specify that so far the recommendation is to perform two consecutive annual Pap smears in the first year of exam, and in case of normal results, once every 3 years…

Line 61: please update this statement as HPV DNA screening is to become available at the Brazilian public health system, as recommended by CONITEC in 2024; add reference below <https://www.gov.br/conitec/pt-br/midias/relatorios/2024/testagem-molecular-para-deteccao-de-hpv-e-rastreamento-do-cancer-do-colo-do-utero/view>

Line 69: please add the word treatment after standard, for clarity.

Line 72: write “healthcare” as one word

Line 75: write “tracking system and management”, instead of tracking management.

Line 76: write new oncology medications in the public healthcare system

Line 77: significant inequalities (plural)

Line 79: write decrease efficiency in detection of…. (not efficacy)

Line 81: …within SUS based on real world evidence…

Line 83: …demographic (singular) characteristics

Line 160: after applying the inclusion and exclusion criteria…

Line 180: highest proportion of advanced disease (not for)

Line 182: The North region had the lowest (not smallest)

Line 267: please verify as the reference shows error, source not found.

Line 299: …determinants of CC burden (not to)

Line 301: for clarity, between are and already add the word diagnosed; with healthcare system

Line 329-30: for clarity, consider editing:…higher mortality rates of CC over time, consistently above the national and other regional trends.

Line 330-31: consider rephrasing: …However, it has been reported that efforts have been made in this region, that increased the population adherence to cervical cancer screening programs. For instance…

Line 333: …replace the word mandatory by necessary: which is necessary to achieve mortality reduction.

Line 339: …data, another Brazilian study…

Line 340: add for clarity …for non-advanced CC stages [11]-

Line 353: rephrase… in patients with advanced disease treated under SUS [15,43,44,46], as observed in this study.

Line 360: …swap letters to read HCUR… , not HRCU.

Line 362-363: this sentence can be deleted because it is out of scope for CC: the statement and the figure of US$ 1.16 trillion refers to all cancers in general, and in addition, the reference is 14 years old, thus the economics is likely different for 2024.

Line 373: add a comma (,) between Hospitalization and length of stay.

Line 379: to be clear and specific, write” …national cervical cancer prevention policies were put in place….”

Line 383: for clarity write…barriers are highlighted and strengthened by the WHO call to eliminate ….

Line 384 : because there are several different HPV vaccines available so far, the manuscript must specify that the “…HPV quadrivalent vaccine has been available in SUS since2014…”

Line 386 to 390: This paragraph should also indicate that among other factors, the impact of vaccination on cervical cancer disease incidence could not be seen so far because the vaccine target population in the last decade was 11-14 y.o. girls (and now also boys), the screening programme starts at 25 y.o. , and the disease appears mostly in advanced aged (e.g. over 50 y.o.). Therefore, there is a need for surveillance of the long term and the heard immunity effects of vaccination, as well as improvements of treatment standards and outcomes in the country.

Line 392: please complement the sentence for clarity.”… half of the women’s records identified through the inclusion criteria….”

Line 394: rephrase “….it is important that staging information be consistently reported in DATASUS…”

Line 399: “Patients may undergo conization procedures in an initial stage, however, they may be included also in SAI-AQ and SIA-AR databases….”

Line 493: …underwent conization in another…”

Line 403 and 405 : …refer to time lag between the diagnosis and the beginning of ….”

Line 404: conization procedures ( not cone…)

Line 414: complement the phrase: …such as increase in coverage of HPV immunization in the target opulation (girls 9-14 y.o. and boys 11-14 y.o.) and more effective screening of women aged 25 to 64….

Line 418: please add the word “equitable” before “access to prevention….

Line 419: you may like to add a final sentence : “This real world data analysis provides evidence for improving the quality of the DATASUS to consistently capturing the CC disease staging for better understanding and resources management, towards achieving the CC elimination goals set by WHO.” Cf. <https://www.who.int/initiatives/cervical-cancer-elimination-initiative>

Line 444: Ref 2, the journal name is missing, cf. Rev Bras. Cancerologia 2023;69(1):2023–5. Cf. <https://rbc.inca.gov.br/index.php/revista/article/view/3700/2644> The reference for incidence with matching figures is also available on an article from INCA cf. <https://www.gov.br/inca/pt-br/assuntos/gestor-e-profissional-de-saude/controle-do-cancer-do-colo-do-utero/dados-e-numeros/incidencia>

Line 445: Ref.3, you need to complete the reference for the mortality of CC in Brazil, or is “Cervical cancer screening, treatment and prophylaxis in Brazil: Current and future perspectives for cervical cancer elimination, by Flávia M. Corrêa, Arn Migowski, Liz M. de Almeida, and Marcelo A. Soares. Front Med (Lausanne). 2022; 9: 945621.Published online 2022 Aug 24. doi: 10.3389/fmed.2022.945621 PMCID: PMC9449345 PMID: 36091685 . Cf. <https://pubmed.ncbi.nlm.nih.gov/36091685/>

Line 447: Ref.4, a more recent reference for the WHO policy on CC could be the original strategic document from 2020, on “Global strategy to accelerate the elimination of cervical cancer as a public health problem” and available online cf. <https://www.who.int/publications/i/item/9789240014107>

Line 452: Ref. 6 is available online at <https://www.saude.sp.gov.br/resources/cve-centro-de-vigilancia-epidemiologica/areas-de-vigilancia/imunizacao/doc/hpv14_informe_tecnico.pdf>

Line 453: Ref. 7 please add link cf. <https://saude.es.gov.br/Media/sesa/Imuniza%C3%A7%C3%A3o/SEI_MS%20-%20Nota%20Informativa%20135%20-%20mudancas%20no%20calendario%20nacional%20de%20vacinacao%202018.pdf>

Line 455: Ref. 8 was published in December 2020 not 2021, cf. <https://pubmed.ncbi.nlm.nih.gov/33331411/>

Line 457: update the reference with the more recent recommendation of the CONITEC report instead cf. Actualize to 2024 cf. <https://www.gov.br/conitec/pt-br/midias/relatorios/2024/testagem-molecular-para-deteccao-de-hpv-e-rastreamento-do-cancer-do-colo-do-utero/view>

Line 572: The World Cancer Report of IARC, in its free online pdf format, has in total 632 pages, cf. <https://publications.iarc.fr/Non-Series-Publications/World-Cancer-Reports/World-Cancer-Report-2014> , and page 632 is the back-cover page with some quotes from well-known scientists or public health leaders, but they do not refer to any expenses or costs of prevention. The word “expensive” appears only four times in the whole book. It may be better to quote chapters 6.2 and 6.3 that elaborate on cancer control and health systems, where costs are discussed.
