# Supplementary material for: Real world data on cervical cancer treatment patterns, healthcare access and resource utilization in the Brazilian public healthcare system
Source: PLoS One. 2024 Oct 30;19(10):e0312757. doi: 10.1371/journal.pone.0312757 (PMC11524504; doi:10.1371/journal.pone.0312757)
Supplement: Attachment — Submitted filename: Response_to_Reviewers.docx [file pone.0312757.s006.docx]

Dear reviewers and Editor,

First, thank you for your comments and insights. Please find below our answers for each point stated, separated per each reviewer and for the additional comments from the editor:

# **REVIEWER 1**

1. **Line 26: “Data from January-2014 until December-2020”. Consider replace ‘until’ with ‘to’.**

Thank you for making this observation. We have updated the manuscript according to your suggestion.

2. **Lines 38-39: “The treatment pattern showed that chemoradiotherapy was the more frequent regimen 39 overall and that the use of chemotherapy increases with staging, as well as the HCRU.”**
   - Suggestion: The treatment pattern showed that chemoradiotherapy was the **most** frequent regimen overall and the use of chemotherapy and HCRU increas**ed** with staging.

Thank you for bringing this to our attention. The sentence was updated according to your suggestion.

3. **Lines 56-57: “has been available for girls from 9 to 14 years since 2014 and 57 for boys from 11 to 14 years since 2017” consider replacing with “has been available for girls aged 9 to 14 e for boys aged 11 to 14” or “has been available for girls from 9 to 14 years old since 2014 and for boys from 11 to 14 years old since 2017 “**

Thank you for the correction. It was updated.

4. **Line 59: “the most effective exam to detect precancerous abnormalities and prevent CC” – reference?**
   - There are recent studies that recognize the HPV DNA or pap smear + HPV DNA as the most effective exam to detect precancerous abnormalities.

We agree with your observation. The focus of the sentence was to describe the recommendation for cervical cancer (CC) screening according to Brazilian guidelines, which still prioritize the cytology (Pap smear) exam. The current guidelines also recommend the HPV DNA test in conjunction with the Pap smear for evaluating women with colposcopy results showing no abnormal findings or only minor abnormalities. In the paragraph, we highlight that the DNA test for HPV screening should be available in the Brazilian public health system by 2024, according to SECTICS/MS Ordinance No. 3 published in March of this year [Line 64].

5. **Line 61: “in case of normal results” – consider replacing with “negative results”. What is a normal result?**

We agree that the information was incorrect. According to Brazilian guidelines for CC screening, the correct term is ‘negative results’ and it was adjusted accordingly.

6. Line 63: “Despite national programs for CC cancer prevention” – the word ‘cancer’ is written twice. ‘CC’ (cervical cancer) and ‘carcer’ right after.

Thank you for your insight. The adjustment has been made in line 63 and throughout the document.

7. **Line 68: I missed mentioning brachytherapy as a line of treatment.**

Thank you for the insight. We agree the information could be more detailed. It was changed: *“For locally advanced/advanced disease (III-IV), the standard is chemoradiotherapy (CCRT), which involves external beam radiation therapy combined platinum-based chemotherapy, followed by brachytherapy.”* [Line 69]

8. **Line 78: “between regions of the country related to social, economic and health access determinants” – perhaps you should also include ‘geographical’**

Thank you for the insight. The term was included in the sentence.

9. **Line 79: “decreased efficacy in the detection of disease” be aware of the semantic differences between effectiveness and efficacy.**

Thank you for bringing this to our attention. We agree and it was already changed to “Efficiency”: “In addition to the delay for inclusion of new oncology medications in public healthcare system, Brazil presents significant inequalities between regions of the country related to social, economic, geographical and health access determinants, which have been associated with decreased efficiency in the detection of disease, treatment adherence and outcomes” [line 91, in the clean version]

10. **Line 80: “To create strategies to reduce the CC burden in Brazil” - It’s more common for native speakers to start a sentence with the gerund as subject than the infinitive form, like “creating strategies to reduce… “**

We appreciate the insight and have updated the sentence accordingly.

11. **Line 90: “SUS is the universal healthcare system of Brazil, which covers 75% of the population [23]”. Is this a necessary information? Is this a precise information? If the SUS is the ‘universal’ healthcare, how can it cover only 75%? SUS is widely available to all the population. It’s a constitutional right. The fact that 24% of users possess private healthcare coverage doesn’t mean they don’t use the system in some way.**

We agree with your consideration. However, it is a common practice to include this information to clarify that the study does not analyze the entire Brazilian population. This is also mentioned in the limitations section, line 391. The sentence in line 93 has been updated to: “*Despite the universal coverage of the Brazilian public healthcare system, approximately 75% rely exclusively on it [23]”.*

12. **Line 105: “Data from January 2014 until December 2020 was gathered of all patients with the 106 International Classification of Diseases tenth revision for cervical cancer (ICD-10 code C53)”. The reference?**
    - **Consider replace ‘until’ with ‘to’.**

Thank you for your comment. The correction has been made and the reference included.

13. **Line 124: Consider replace ‘until’ with ‘to’. You have already said the study’s period. Maybe you could replace the ‘data from … to… ‘ with another expression.**

The expression “From january 2014 to December 2020” was replaced for “during the study period” [line 125].

14. **Line 130: “Patient’s stages were considered in the first ICD-10 claim in SIA-AQ and SIA-AR databases (subsets of chemotherapy and radiotherapy from SIA database), which provides stage information.” In the same sentence, you have said that those databases provide stage information twice. Also, you have said that previously in lines 102-103.**

Thank you for your comment. The duplicated information was removed, and the paragraph adjusted.

15. **Lines 130-132: “Patients with stage inconsistent information (i.e., stage information different from I to IV) at first cervical cancer claim were excluded (Fig 1)” – It would be better written as ‘patients with inconsistent stage information…’**

Thank you. The sentence was updated.

16. **Line 139: “Additionally, in the staged patient’s distance”. Take the ‘in’ word out.**

Thank you. The sentence was updated.

17. **Lines 140-142: “(measured as the date of treatment initiation, defined in this study as the time elapsed from the first claim of ICD142 10 C53 to first treatment procedure [CT, RT or surgery – except conization/cone biopsy])”.**
    - **Consider removing the ‘measured as the date of treatment initiation’ and just use the ‘defined in this study…’. They explain the same thing, it’s repetitive.**

Thank you for your insight. It was adjusted.

18. **Lines 149-150: “with absolute frequencies and percentages with 95% confidence interval (CI) and percentages”. You’ve written ‘percentages’ twice.**

Thank you. The second word was excluded.

19. **Lines 153-154: “Thus, the longer the follow-up, lower the PPPM will be, even if there are many procedures claims”. You don’t need the ‘will be’, because you are already using a comparative form.**

Thank you for your insight. It was adjusted.

20. **Line 157: “The number of LOTs received was summarized.” – I understand this information was already stated in the previous sentence “…and most frequently reported treatments for each line of treatment (LOT) using a Sankey diagram”.**

Thank you for making this observation. We agree it was stated previously. The sentence was excluded.

21. **Are the abbreviations ‘pppm’ and ‘hcru’ official or used in other reference? They’re confusing and difficult to associate along the reading. I had to return to the Methods section all the time to understand them.**

The acronym HCRU is widely used to designate Healthcare Resources Utilization, especially in studies conducted using secondary databases or medical records, so we chose to keep it in the text.

As we understand that PPPM may not be so common and may be confused with other acronyms, we chose to replace it throughout the text with "per patient per month". We kept the acronym only in the tables, with its respective legend below.

22. Table 1
    - **In the title: ‘Description of sociodemographic characteristics of included patients’ instead of ‘(…)patients included’.**

It was adjusted. Thank you.

- **In ‘Ethnicity N(%)’ the percentage and the absolute numbers doesn’t match with the total included population (206,861). After several calculations, I concluded that the percentages were calculated after the ‘all patients’ minus ‘missing information’ and that’s not clear in the text. Meaning, in this case, the total N is not 206,861, but 178,582. Is that correct? Perhaps you could add an asterisk to clear that data like you did in ‘outpatient visit’ in table 2.**

Thank you for bringing this to our attention. We believe the correct percentage should be calculated based on the number of each ethnicity relative to the total population (206,861). The percentages have been adjusted accordingly.

- **You have already said in the Methods section about the age at index date, and in the Results section about the total of staged patients. Is it necessary to write that again in the table?**

We agree this information is clear throughout the manuscript and removed it from the table.

23. Table 2:
    - **Hospitalization rate in days???**

Hospitalization was measured as the mean or median number of hospitalizations per patient during the study period. In the Median per patient per month, it is possible to analyze the median number of hospitalizations per patient each month. Then, the Length of Stay (LOS) measures the mean and median number of days hospitalized.

- “**Outpatient visit^1^” – What does that superscripted number refer to?**

Thank you for the observation. This number was not related to any information, and it was removed.

- **Line 201: “* Includes all patients with stage 1-4, and those non-staged.”. I didn’t find any asterisk in the table. I guess this note refers to the “Outpatient visit^1^”.**

Thank you for the observation. The asterisk “*” belongs to the column “All patients.” The legend for Table 2 clarifies why the sum of the staged patient columns (Stages I-IV = 90,073) does not match the column “All patients” (206,861). The column “All patients” includes all patients with the ICD-10 code C53 in the database, both staged and those with no stage information available. I updated table 1 to have the same asterisk pattern as Table 2.

- **Is there any information/data on brachytherapy?**

Information on the frequency of brachytherapy use is detailed in the Supplementary Material (Table S3).

- **You have already said in the Results section about the total of staged patients. Is it necessary to write that again in the table?**

We agree that the information was already mentioned. However, we prefer to keep the legend in these tables to clarify why the sum of the staged patient columns (Stages I-IV = 90,073) does not match the column “All patients” (206,861). The column “All patients” includes all patients with the ICD-10 code C53 in the database, both staged and those with no stage information available.

24. **Line 199-200: “Legend: LOS: length of stay; IQR: interquartile range; CI: confidence interval; SD: standard 200 deviation; PPPM: per patient per month.” Those abbreviations were already explained previously**.

We appreciate your suggestion. To facilitate readability, we have opted to include this information in the legend.

25. **Line 250 and S2 table: “Patients with stage III and IV have traveled a greater median distance of 7.96 km [IQR 0.0 - 89.16] compared to 4.88 km 252 [IQR 0.0 - 75.16] in the non-advanced stages (S2 Table)” However, in the Supplementary table, ‘4.88 km 252 [IQR 0.0 - 75.16]’ refers to advanced stage IV. You should review the table later.**

Thank you for bringing this to our attention. The table was reviewed, and the sentence updated: “*Patients with stage III and IV have traveled a greater median distance of 7.96 km [IQR 0.0 - 89.16] and 4.88 km [IQR 0.0 - 75.16], respectively, compared to the non-advanced stages”*

26. **S2 Table: The stratification per kilometers is the sum of all the kilometers spent traveling by all the patients on all the visits, correct? I took some time to get that.**

The stratification by kilometers indicates the number of visits to the health center categorized by distance. The percentage reflects the proportion of the total number of visits. This information is detailed in the table legend and has been adjusted for clarity.

27. **S3 Table:**
    - **‘Surgery^3^ - N (%)’ – What does the superscripted number stand for?**

Thank you for bringing this to our attention. The information was missing. It refers to the statement that ‘Patients may undergo multiple surgical procedures,’ which has now been included in the table legend.

- **You don’t need the comma in the sentence ‘Cervical cancer treatment, according to disease stage.’**

It was excluded. Thank you.

28. **Line 259: ‘Lymphadenectomy with pelvic lymphadenectomy’. Try to replace for only ‘pelvic lymphadenectomy’’**

Thank you for your insight. It was adjusted.

29. **Line 268: “About 90% of stage I patients versus 67% of stage IV patients were submitted to RT (Error! Reference source not found.).” I don’t think there’s any reference in there.**

Thank you for your comment. This reference refers to ‘Figure 4B’. It was linked automatically by Word’s ‘Cross-reference’ feature, which likely contained an error. It has now been updated according to PLOS ONE guidelines.

30. **Line 295: Avoid using the first-person plural. Suggestion: ‘so far’.**

Thank you for the insight. It was adjusted.

31. **Line 297: ‘Although there were improvements in the prevention of cervical cancer in Brazil’. The prevention policies continue to improve, perhaps you should write as: “Although there have been improvements… “**

Thank you for the insight. It was adjusted.

32. **Line 299: “Like our results” –Avoid using the first-person plural. Suggestion: ‘****Similar to these findings, other Brazilian studies’**

Thank you for the insight. The sentence was changed: *“Similar to the findings of this study, other Brazilian studies reported a median age of 50 years in CC patients…”*

33. **Line 300: mean or median?**

Thank you for your comment. The information refers to the mean age found in other Brazilian studies and has been adjusted accordingly

34. **Line 301: ‘were’ not ‘are’**

It was adjusted. Thank you.

35. **Line 324: Replace ‘in most wealthy’ with ‘in** **wealthier.’**

It was adjusted. Thank you.

36. **Line 324: “what was mentioned about health access and population’s well-being” – mentioned where? What about health access and population’s well-being?**

Thank you for your observation. We were referring to the previous paragraphs. We agree it was not evident and it was adjusted: *“The higher concentration of patients in wealthier Brazilian regions reinforces the relationship between greater access to healthcare and earlier disease diagnosis, which can result in better outcomes.”*

37. **Line 325-326: “as with higher income per-capita” – ‘per-capita income’**

It was adjusted. Thank you.

38. **Lines 325-327: “since these regions are characterized as with higher income per-capita, educational levels, concentration of health services, access to reference medical units and higher health resources per-capita in both private and public sectors in these regions” – ‘these regions’ is mentioned twice in the same sentence. Perhaps you should consider turn this sentence into two different ones, so it doesn’t get confused.**

Thank you for the insight. We agree the sentence was loo long, and it has been adjusted: *“The higher concentration of patients in wealthier Brazilian regions reinforces the relationship between greater access to healthcare and earlier disease diagnosis, which can result in better outcomes [32]. These areas are characterized by higher per-capita income, elevated educational levels, concentration of health services, access to reference medical units, and higher per-capita health resources in both private and public sectors [32]”*

39. **‘higher health resources per-capita’ 🡪 ‘higher per-capita health resources’**

It has been updated. Thank you.

40. **Lines 336-339: Suggestion: “Even though it has been mandatory for all cancer patients to start treatment within 60 days from the** **confirmed histological diagnosis, this analysis shows that it is not yet a reality for all patients diagnosed with CC”.**

Thank you for your suggestion. It has been adjusted.

41. **Lines 351-354: This is confusing – “In patients with recurrent, persistent, or metastatic disease paclitaxel regimens, in addition to cisplatin and other drugs, and associated to RT, are usually used in the clinical practice in patients with advancing disease treated under SUS, which was observed in this study”**
    - **Suggestion: In patients with recurrent, persistent, or metastatic disease paclitaxel regimens** **associated to cisplatin or other drugs and RT are commonly used in the clinical practice, as observed in this study.**

Thank you for your suggestion. We agree it was not clear, and it was adjusted: *“In clinical practice, patients with advanced disease treated in SUS generally receive paclitaxel regimens associated to cisplatin and other drugs, along with RT [15,43,44,46], as observed in this study.”*

42. **Lines 355-358: The verb tenses are confused in this sentence, because It’s a finished action in a specific time of the past, so we use the simple past not the present perfect. “In 2021, however, new evidence has shown that adjuvant chemotherapy given after standard cisplatin-based chemoradiation for women with locally advanced CC has no effect in overall survival or progression free survival, changing the management of the disease”**
    - **Suggestion: In 2021, however, new evidence showed that adjuvant chemotherapy given after standard cisplatin-based chemoradiation for women with locally advanced CC had no effect in overall survival or progression free survival, changing the management of the disease.**

Thank you for the suggestion. It was adjusted accordingly.

43. **Line 371: Avoid using the first-person plural – Suggestion: ‘Although this study…’**

It was updated. Thank you.

44. **Lines 384-390: “. Furthermore, the HPV vaccine has been available in SUS since 2014, initially for girls aged 385 11 to 13 years, with a gradual expansion to other age groups, being currently recommended for 386 girls in the age group from 9 to 14 years, and for boys aged 11 to 14 years. However, immediate effects of HPV vaccination could not be seen since the target population is consisted of younger age groups and the disease usually appears with the advancing age, indicating the need of surveillance of the long-term effects and herd immunity, and an improvement of treatment standards and outcomes in the country”**
    - **You have mentioned this vaccine information previously in the Background section. It’s not necessary repeat it.**
    - **“Could” is commonly used to describe potential or hypothetical situations. I don’t believe it fits in this sentence very well**
    - **Suggestion: Despite HPV vaccine has been available in SUS since 2014, its immediate effects cannot yet be observed, as the target population consists of younger age groups, and the disease usually appears later in life, indicating the need for effects and herd immunity long-term surveillance, as well as improvements in treatment standards and outcomes in the country.**

Thank you for your insights. We updated the paragraph based on your suggestion.

45. **Line 399-400 – Suggestion: “Patient may undergo to Conization procedure in an initial stage; however, he’s only included in SIA-AQ and SIA-AR databases after an advanced stage diagnosis.”**

Thank you for your suggestion. The sentence was adjusted.

46. **Lines 408-409: It’s not correct to assume that private healthcare users do not use the public health system. We don’t know for sure the percentage of the Brazilian population that certainly use the public health system. So, you’d better change this last sentence.**

We agree with your consideration. However, it is a common practice to include this information to clarify that the study does not analyze the entire Brazilian population. Data on the number of people who rely exclusively on SUS was extracted from the National Supplementary Health Agency (ANS). The sentence was changed to: “*It also important to mention that the healthcare resource utilization related to CC does not represent the entire Brazilian population, as approximately 75% rely exclusive on it [23]”* [Line 391].

47. **In the limitations setting, probably you should write about the fact that those are secondary data and the underreporting of data related to the database in a country of continental dimensions and with significant deficiencies in the public health system. In addition, you should highlight about the need for more complete and efficient database, as well as the need for better methods to collect and work this type of data, given its importance for public health service.**

Thank you for making this observation. We agree this information was really missing and we have already included it: *“The major limitation of retrospective data is the probability of incomplete data, and the variable quality of non-mandatory data, which is intrinsic to retrospective database studies. Additionally, the databases used in this study are administrative and reimbursement-oriented, and therefore lack detailed, granular clinical data. This underscores the need for more complete and efficient methods for collecting and managing with this type of data, given its significance for understanding population characteristics.”* [Line 385]

# **REVIEWER 2**

**The manuscript by Campuzano and collaborators, presenting an analysis on “Real world data on cervical cancer treatment patterns, healthcare access and resource utilization in the Brazilian public health system”, deserves publication.**

In general, the manuscript provides an interesting and useful analysis of a large population, derived from the Brazilian national data, based on healthcare records accumulated over 7 years (2014-2020), and offers evidence to refining the public health policy regarding access to cervical cancer treatment, lines of treatment, and resources utilization.

Still, the paper can be further improved for clarity by amendments in the text (and grammar). Also the references need further attention to quoting more recent, more relevant documents, and for accuracy, in quoting completed references, as some references lack journal names or records, such as page numbers, or sometimes quote the wrong page number. Please add the links to the webpages (url) where the references can be easily accessed, as possible.

Thank you for your observation. The references have been reviewed and updated.

Specific points for improvements:

**Line 49: the number of 6,627 deaths cannot be found in the “Brasil. Ministério da saúde. Instituto Nacional de Câncer. Estimativa de Câncer no Brasil. 2020”, but rather on a news article of 2023 available at** [**https://agenciabrasil.ebc.com.br/saude/noticia/2023-07/diagnosticos-de-cancer-no-colo-do-utero-devem-somar-17-mil-ate-2025**](https://agenciabrasil.ebc.com.br/saude/noticia/2023-07/diagnosticos-de-cancer-no-colo-do-utero-devem-somar-17-mil-ate-2025) **.**

**However, the scientific reference mentioning the number of 6,627 deaths can be found on the article “Cervical cancer screening, treatment, and prophylaxis in Brazil: Current and future perspectives for cervical cancer elimination. Flávia M. Corrêa, Arn Migowski, Liz M. de Almeida, and Marcelo A. Soares. Frontiers of Medicine (Lausanne). 2022; 9: 945621.Published online 2022 Aug 24. doi: 10.3389/fmed.2022.945621 PMCID: PMC9449345 PMID: 36091685**

**I invite the authors to ascertain and validate the source of data, and replace reference 3 by F Correa et al. 2022.**

Thank you for bringing this to our attention. We agree the references were not directly related to data, and it is already adjusted. Expected incidence for each year of the 2020-2022 triennium is 16,590 is available in “Instituto Nacional de Câncer José Alencar Gomes da Silva. Estimativa 2020 : incidência de câncer no Brasil / Instituto Nacional de Câncer José Alencar Gomes da Silva. – Rio de Janeiro : INCA, 2019. https://www.inca.gov.br/sites/ufu.sti.inca.local/files/media/document/estimativa-2020-incidencia-de-cancer-no-brasil.pdf”

For the number of deaths (6,627), we updated the reference for the manuscript that you shared as well as for the panel data source from which it was extracted: https://www.inca.gov.br/app/mortalidade The number of deaths 6,627)

**Line 50: replace the word “expected” by “reported” for 2020, as the number of deaths was so reported in the above reference.**

The sentence was adjusted to: *“In Brazil, the estimated incidence for each year of the 2020-2022 triennium is 16,590, with 6,627 deaths related to the disease reported in 2020.”* Thank you.

**Line 50: I could not find the number of “20.48” on the paper by Santos MDO, Fernando J, Oliveira P, Maria L, Almeida D, Cancela MDC. Estimativa de Incidência de Câncer no Brasil, 2023-2025.**

**It seems that 20.48 cases per 100.000 women relates to incidence - not prevalence - of type of cancer: the publication mentioning this figure is by Flavia Correa et al., 2022. This number can also be found on the online article of INCA of 2023, please see reference below: harmonize references accordingly.** [**https://www.gov.br/inca/pt-br/assuntos/gestor-e-profissional-de-saude/controle-do-cancer-do-colo-do-utero/dados-e-numeros/incidencia**](https://www.gov.br/inca/pt-br/assuntos/gestor-e-profissional-de-saude/controle-do-cancer-do-colo-do-utero/dados-e-numeros/incidencia)

Thank you for your comment. We agree with your observations and have adjusted the sentence accordingly. The estimative indeed refers to incidence and is reported in the official source of Brazilian National Cancer Institute (INCA): Instituto Nacional de Câncer (Brasil). Estimativa 2023 : incidência de câncer no Brasil / Instituto Nacional de Câncer. – Rio de Janeiro : INCA, 2022 <https://www.inca.gov.br/sites/ufu.sti.inca.local/files/media/document/estimativa-2023.pdf>

**Line 52: as mentioned above, I suggest indicate reference 3 (as above), not 2.**

The paragraph and references were updated according to your suggestions. Thank you.

**Line 53: please update the WHO statement and mention that WHO** **adopted in 2020 the “Global strategy to accelerate the elimination of cervical cancer as a public health problem”, based on three pillars : vaccination, screening, and treatment, as priority to reduce morbidity and mortality, as well as costs related to cervical cancer.**

Thank you for your suggestion. The paragraph was adjusted, and the reference updated accordingly.

**Line 55: the better and more recent reference no. 4 should be the WHO Global strategy to accelerate the elimination of cervical cancer as a public health problem. 2020, cf.** [**https://www.who.int/publications/i/item/9789240014107**](https://www.who.int/publications/i/item/9789240014107)

The reference was updated accordingly.

**Line 57: please add one sentence to clarify that today HPV vaccination coverage in Brazil remains low,** **as only less than 10% of young women 19-24 years old have been vaccinated.**

We appreciate your comment and inform you that the sentence has been updated. However, we apologize for not mentioning the data cited as we did not find any reference that indicates the same values.

**Line 58: rephrase for clarity that In Brazil until today the CC screening program has been based on cytology by Papanicolau smear (Pap smear), for women aged 25 to 64 years old.**

Thank you for your insight. We agree this information was really missing and already included it.

**Line 60: please specify that so far the recommendation is to perform two consecutive annual Pap smears in the first year of exam, and in case of normal results, once every 3 years…**

The information was included in the sentence. Thank you.

**Line 61: please update this statement as HPV DNA screening is to become available at the Brazilian public health system, as recommended by CONITEC in 2024; add reference below** [**https://www.gov.br/conitec/pt-br/midias/relatorios/2024/testagem-molecular-para-deteccao-de-hpv-e-rastreamento-do-cancer-do-colo-do-utero/view**](https://www.gov.br/conitec/pt-br/midias/relatorios/2024/testagem-molecular-para-deteccao-de-hpv-e-rastreamento-do-cancer-do-colo-do-utero/view)

Thank you for your suggestion. We agree, and it was already included in the text: “*According to the National Commission for the Incorporation of Technologies (CONITEC) recommendation, HPV DNA screening is set to become available in the Brazilian public health system in 2024*” [Line 63].

**Line 69: please add the word treatment after standard, for clarity.**

Thank you for the comment. The word was included.

**Line 72: write “healthcare” as one word**

It was adjusted. Thank you.

**Line 75: write “tracking system and management”, instead of tracking management.**

It was adjusted. Thank you.

**Line 76: write new oncology medications in the public healthcare system**

It was adjusted. Thank you.

**Line 77: significant inequalities (plural)**

It was adjusted. Thank you.

**Line 79: write decrease** **efficiency in detection of…. (not efficacy)**

Thank you for bringing this to our attention. The word was already changed.

**Line 81: …within SUS based on real world evidence…**

It was adjusted. Thank you.

**Line 83: …demographic (singular) characteristics**

It was adjusted. Thank you.

**Line 160: after** **applying the inclusion and exclusion criteria…**

It was adjusted. Thank you.

**Line 180: highest proportion of advanced disease (not for)**

It was adjusted. Thank you.

**Line 182: The North region had the lowest (not smallest)**

It was adjusted. Thank you.

**Line 267: please verify as the reference shows error, source not found.**

Thank you for your comment. This reference refers to ‘Figure 4B’. It was linked automatically by Word’s ‘Cross-reference’ feature, which likely contained an error. It has now been updated according to PLOS ONE guidelines.

**Line 299: …determinants of CC burden (not to)**

It was adjusted. Thank you.

**Line 301: for clarity, between are and already add the word diagnosed; with healthcare system**

The sentence was adjusted according to your suggestion. Thank you.

**Line 329-30: for clarity, consider editing:…higher mortality rates of CC over time, consistently above the national and other regional trends.**

Thank you for the suggestion. The sentence was adjusted accordingly.

**Line 330-31: consider rephrasing: …However, it has been reported that efforts have been made in this region, that increased the population adherence to cervical cancer screening programs. For instance…**

We agree the sentence was not clear. It was adjusted according to your suggestion. Thank you.

**Line 333: …replace the word mandatory by necessary: which is necessary to achieve mortality reduction.**

The word was changed. Thank you.

**Line 339: …data, another Brazilian study…**

The word was changed. Thank you.

**Line 340: add for clarity …for non-advanced CC stages [11]-**

The word was included. Thank you.

**Line 353: rephrase… in patients with advanced disease treated under SUS [15,43,44,46], as observed in this study.**

Thank you for your comment. The sentence was rephrased and your suggestion included: *“In clinical practice, patients with advanced disease treated in SUS generally receive paclitaxel regimens associated to cisplatin and other drugs, along with RT [15,43,44,46], as observed in this study” [Line351]*

**Line 360: …swap letters to read HCUR… , not HRCU.**

The letters were adjusted. Thank you for the observation.

**Line 362-363: this sentence can be deleted because it is out of scope for CC: the statement and the figure of US$ 1.16 trillion refers to all cancers in general, and in addition, the reference is 14 years old, thus the economics is likely different for 2024.**

Thank you for your insight. We agree that the reference is too old. The sentence was removed.

**Line 373: add a comma (,) between Hospitalization and length of stay.**

Comma was included. Thank you.

**Line 379: to be clear and specific, write” …national cervical cancer prevention policies were put in place….”**

Thank you for your insight. The sentence was adjusted accordingly.

**Line 383: for clarity write…barriers are highlighted and strengthened by the WHO call to eliminate ….**

It was updated according to your suggestion. Thank you.

**Line 384 : because there are several different HPV vaccines available so far, the manuscript must specify that the “…HPV quadrivalent vaccine has been available in SUS since2014…”**

We agree it would be better to specify it. Thank you for your suggestion.

**Line 386 to 390: This paragraph should also indicate that among other factors, the impact of vaccination on cervical cancer disease incidence could not be seen so far because the vaccine target population in the last decade was 11-14 y.o. girls (and now also boys), the screening programme starts at 25 y.o. , and the disease appears mostly in advanced aged (e.g. over 50 y.o.). Therefore, there is a need for surveillance of the long term and the heard immunity effects of vaccination, as well as improvements of treatment standards and outcomes in the country.**

Thank you for your observation. The paragraph was updated: *“Despite, HPV quadrivalent vaccine has been available in SUS since 2014, its immediate effects cannot yet be observed. While the vaccine targets younger age groups, the screening program usually starts at 25 years old, and the disease mostly appears in advancing age, over 50 years old [56–58]. Therefore, there is a need for long-term surveillance of herd immunity effects, as well as improvements in treatment standards and outcomes in the country.”* [Line 381].

**Line 392: please complement the sentence for clarity.”… half of the women’s records identified through the inclusion criteria….”**

Thank you for your suggestion. It was adjusted accordingly.

**Line 394: rephrase “….it is important that** **staging information be consistently reported in DATASUS…”**

The information was included. Thank you.

**Line 399: “Patients may undergo conization procedures in an initial stage, however, they may be included also in SAI-AQ and SIA-AR databases….”**

The sentence was adjusted. Thank you.

**Line 493: …underwent conization in another…”**

The sentence was adjusted. Thank you.

**Line 403 and 405 : …refer to time lag between the diagnosis and the beginning of ….”**

The information was included. Thank you.

**Line 404: conization procedures ( not cone…)**

The word was adjusted. Thank you.

**Line 414: complement the phrase: …such as increase in coverage of HPV immunization in the target opulation** **(girls 9-14 y.o. and boys 11-14 y.o.) and more effective screening of women aged 25 to 64….**

The information was included. Thank you.

**Line 418: please add the word “equitable” before “access to prevention….**

The word was included. Thank you.

**Line 419: you may like to add a final sentence : “This real world data analysis provides evidence for improving** **the quality of the DATASUS to consistently capturing the CC disease staging for better understanding and resources management,** **towards achieving the CC elimination goals set by WHO.” Cf.** [**https://www.who.int/initiatives/cervical-cancer-elimination-initiative**](https://www.who.int/initiatives/cervical-cancer-elimination-initiative)

Thank you for your suggestion. We agree that this information is important to be included in the conclusion. I was included in line 420: “Additionally, this real world analysis highlights the need to improve the quality of DATASUS to consistently capture the CC disease staging. Such improvements are essential for better understanding and resource management, towards achieving the WHO’s CC elimination goals.”

**Line 444: Ref 2, the journal name is missing, cf. Rev Bras. Cancerologia 2023;69(1):2023–5. Cf.** [**https://rbc.inca.gov.br/index.php/revista/article/view/3700/2644**](https://rbc.inca.gov.br/index.php/revista/article/view/3700/2644) **The reference for incidence with matching figures is also available on an article from INCA cf.** [**https://www.gov.br/inca/pt-br/assuntos/gestor-e-profissional-de-saude/controle-do-cancer-do-colo-do-utero/dados-e-numeros/incidencia**](https://www.gov.br/inca/pt-br/assuntos/gestor-e-profissional-de-saude/controle-do-cancer-do-colo-do-utero/dados-e-numeros/incidencia)

Thank you. The references have been updated and corrected.

**Line 445: Ref.3, you need to complete the reference for the mortality of CC in Brazil, or is “Cervical cancer screening, treatment and prophylaxis in Brazil: Current and future perspectives for cervical cancer elimination, by Flávia M. Corrêa, Arn Migowski, Liz M. de Almeida, and Marcelo A. Soares. Front Med (Lausanne). 2022; 9: 945621.Published online 2022 Aug 24. doi: 10.3389/fmed.2022.945621 PMCID: PMC9449345 PMID: 36091685 . Cf.** [**https://pubmed.ncbi.nlm.nih.gov/36091685/**](https://pubmed.ncbi.nlm.nih.gov/36091685/)

Thank you. The references have been updated and corrected.

**Line 447: Ref.4, a more recent reference for the WHO policy on CC could be the original strategic document from 2020, on “Global strategy to accelerate the elimination of cervical cancer as a public health problem” and available online cf.** [**https://www.who.int/publications/i/item/9789240014107**](https://www.who.int/publications/i/item/9789240014107)

Thank you. The references have been updated and corrected.

**Line 452: Ref. 6 is available online at** [**https://www.saude.sp.gov.br/resources/cve-centro-de-vigilancia-epidemiologica/areas-de-vigilancia/imunizacao/doc/hpv14_informe_tecnico.pdf**](https://www.saude.sp.gov.br/resources/cve-centro-de-vigilancia-epidemiologica/areas-de-vigilancia/imunizacao/doc/hpv14_informe_tecnico.pdf)

Thank you. The references have been updated and corrected.

**Line 453: Ref. 7 please add link cf.** [**https://saude.es.gov.br/Media/sesa/Imuniza%C3%A7%C3%A3o/SEI_MS%20-%20Nota%20Informativa%20135%20-%20mudancas%20no%20calendario%20nacional%20de%20vacinacao%202018.pdf**](https://saude.es.gov.br/Media/sesa/Imuniza%C3%A7%C3%A3o/SEI_MS%20-%20Nota%20Informativa%20135%20-%20mudancas%20no%20calendario%20nacional%20de%20vacinacao%202018.pdf)

Thank you. The references have been updated and corrected.

**Line 455: Ref. 8 was published in December 2020 not 2021, cf.** [**https://pubmed.ncbi.nlm.nih.gov/33331411/**](https://pubmed.ncbi.nlm.nih.gov/33331411/)

Thank you. The references have been updated and corrected.

**Line 457: update the reference with the more recent recommendation of the CONITEC report instead cf. Actualize to 2024 cf.** [**https://www.gov.br/conitec/pt-br/midias/relatorios/2024/testagem-molecular-para-deteccao-de-hpv-e-rastreamento-do-cancer-do-colo-do-utero/view**](https://www.gov.br/conitec/pt-br/midias/relatorios/2024/testagem-molecular-para-deteccao-de-hpv-e-rastreamento-do-cancer-do-colo-do-utero/view)

Thank you. The references have been updated and corrected.

**Line 572: The World Cancer Report of IARC, in its free online pdf format, has in total 632 pages, cf.** [**https://publications.iarc.fr/Non-Series-Publications/World-Cancer-Reports/World-Cancer-Report-2014**](https://publications.iarc.fr/Non-Series-Publications/World-Cancer-Reports/World-Cancer-Report-2014) **, and page 632 is the back-cover page with some quotes from well-known scientists or public health leaders, but they do not refer to any expenses or costs of prevention. The word “expensive” appears only four times in the whole book. It may be better to quote chapters 6.2 and 6.3 that elaborate on cancer control and health systems, where costs are discussed.**

Thank you. The references have been updated and corrected.

Additional Editor comments:

Journal Requirements:

**When submitting your revision, we need you to address these additional requirements.**

**1. Please ensure that your manuscript meets PLOS ONE's style requirements, including those for file naming. The PLOS ONE style templates can be found at** [**https://journals.plos.org/plosone/s/file?id=wjVg/PLOSOne_formatting_sample_main_body.pdf**](https://journals.plos.org/plosone/s/file?id=wjVg/PLOSOne_formatting_sample_main_body.pdf) **and** [**https://journals.plos.org/plosone/s/file?id=ba62/PLOSOne_formatting_sample_title_authors_affiliations.pdf**](https://nam04.safelinks.protection.outlook.com/?url=https%3A%2F%2Fjournals.plos.org%2Fplosone%2Fs%2Ffile%3Fid%3Dba62%2FPLOSOne_formatting_sample_title_authors_affiliations.pdf&data=05%7C02%7Cpaula.pungartnik%40iqvia.com%7Cbd7725ec0a664382979508dcd697973b%7C5989ece0f90e40bf9c791a7beccdb861%7C0%7C0%7C638621195291960923%7CUnknown%7CTWFpbGZsb3d8eyJWIjoiMC4wLjAwMDAiLCJQIjoiV2luMzIiLCJBTiI6Ik1haWwiLCJXVCI6Mn0%3D%7C0%7C%7C%7C&sdata=Op%2BUnZ1PhR0dHDhGjdJ3iFL6GCJ3kVQhDxOQm62FNpI%3D&reserved=0)

Documents revised and adjusted according to PLOS ONE's Guidelines.

2. **We note that the grant information you provided in the ‘Funding Information’ and ‘Financial Disclosure’ sections do not match.**

**When you resubmit, please ensure that you provide the correct grant numbers for the awards you received for your study in the ‘Funding Information’ section.**

We apologize for the misunderstanding. The information was adjusted.

3. **Thank you for stating the following in the Competing Interests section:**

**“I have read the journal's policy and the authors of this manuscript have the following competing interests: TMFC, MACSM, PMB, MACR, MN, LPLG and CPM are employees of Merck Sharp Dohme Farmacêutica Ltda, Sao Paulo, Brazil, who may own stock and/or hold stock options in Merck & Co., Inc., Rahway, NJ, USA. ANR is a scientific medical consultant who was paid by MSD Brazil. PCP and ACS were employees of IQVIA Brazil, which was contracted by MSD Brazil to conduct the study.”**

**Please confirm that this does not alter your adherence to all PLOS ONE policies on sharing data and materials, by including the following statement: "This does not alter our adherence to  PLOS ONE policies on sharing data and materials.” (as detailed online in our guide for authors** [**http://journals.plos.org/plosone/s/competing-interests**](http://journals.plos.org/plosone/s/competing-interests)**).  If there are restrictions on sharing of data and/or materials, please state these. Please note that we cannot proceed with consideration of your article until this information has been declared.**

**Please include your updated Competing Interests statement in your cover letter; we will change the online submission form on your behalf.**

We declare that there are no competing interests that could interfere with the full and objective presentation, peer review, editorial decision-making, or publication of this research. The role of each author was described to be transparent with each competing interest statement as per PLOS ONE guidelines. Competing Interests statement was included in the cover letter with the sentence “This declaration does not alter our adherence to PLOS ONE policies on sharing data and materials.”

Please, do not hesitate to contact me if you have any questions.

4. **Please upload a new copy of Figure 5 as the detail is not clear. Please follow the link for more information:** [**https://blogs.plos.org/plos/2019/06/looking-good-tips-for-creating-your-plos-figures-graphics/**](https://blogs.plos.org/plos/2019/06/looking-good-tips-for-creating-your-plos-figures-graphics/)**"** [**https://blogs.plos.org/plos/2019/06/looking-good-tips-for-creating-your-plos-figures-graphics/**](https://nam04.safelinks.protection.outlook.com/?url=https%3A%2F%2Fblogs.plos.org%2Fplos%2F2019%2F06%2Flooking-good-tips-for-creating-your-plos-figures-graphics%2F&data=05%7C02%7Cpaula.pungartnik%40iqvia.com%7Cbd7725ec0a664382979508dcd697973b%7C5989ece0f90e40bf9c791a7beccdb861%7C0%7C0%7C638621195291972926%7CUnknown%7CTWFpbGZsb3d8eyJWIjoiMC4wLjAwMDAiLCJQIjoiV2luMzIiLCJBTiI6Ik1haWwiLCJXVCI6Mn0%3D%7C0%7C%7C%7C&sdata=wTqECES60UA4aHBDm%2FmrOMTFqFuJdgc7OrKgk1eVD%2Fw%3D&reserved=0)**"**

We apologize for Figure 5 not being up to the journal's standards. Because this figure is composed of 4 Sankey diagrams, which gives it a lot of information, we decided to split the old Figure 5 into two new Figures (New Fig. 5 and Fig. 6).

So in this new version, Fig. 5 includes the Sankey diagram of treatment patterns for cervical cancer Stage I (Fig. 5A) and Stage II (Fig. 5B) and Fig. 6 includes the Sankey diagram of treatment patterns for cervical cancer Stage III (Fig. 6A) and Stage IV (Fig. 6B).

We also inform you that we submitted both figures to the PACE platform, as recommended by PlosOne.

**5. Please review your reference list to ensure that it is complete and correct. If you have cited papers that have been retracted, please include the rationale for doing so in the manuscript text, or remove these references and replace them with relevant current references. Any changes to the reference list should be mentioned in the rebuttal letter that accompanies your revised manuscript. If you need to cite a retracted article, indicate the article’s retracted status in the References list and also include a citation and full reference for the retraction notice.**

Thank you for your observation. We inform you that all references in the manuscript have been updated and/or revised and corrected.

**Additional Editor Comments:**

**The manuscript presents valuable insights based on national Brazilian data. The analysis encompasses a large population, offering findings that are highly relevant to public health policies. However, the paper would benefit from a thorough language review to enhance sentence structure and correct grammatical errors. Additionally, the references should be revised to ensure compliance with the journal's formatting guidelines.**

We really appreciate the reviewers’ comments and suggestions. The manuscript was revised and adjusted accordingly.

Thank you very much, we really appreciated your comments, and we hope that the revised version will be considered suitable for publication by the journal.
